# The mothers, Omega-3 and mental health study

**DOI:** 10.1186/1471-2393-11-46

**Published:** 2011-06-22

**Authors:** Ellen Mozurkewich, Julie Chilimigras, Chelsea Klemens, Kristie Keeton, Lucy Allbaugh, Susan Hamilton, Deborah Berman, Delia Vazquez, Sheila Marcus, Zora Djuric, Anjel Vahratian

**Affiliations:** 1 University of Michigan, Department of Obstetrics and Gynecology, Division of Maternal-Fetal Medicine, Ann Arbor, Michigan, USA; 2 Michigan State University College of Human Medicine, East Lansing, Michigan, USA; 3 Integrated Health Associates, Ann Arbor, Michigan, USA; 4 University of Michigan, Department of Psychiatry, Ann Arbor, Michigan, USA; 5 University of Michigan, Department of Pediatrics & Communicable Diseases, Ann Arbor, Michigan, USA; 6 University of Michigan, Department of Family Medicine, Ann Arbor, Michigan, USA

## Abstract

**Background:**

Major depressive disorder (MDD) during pregnancy and postpartum depression are associated with significant maternal and neonatal morbidity. While antidepressants are readily used in pregnancy, studies have raised concerns regarding neurobehavioral outcomes in exposed infants. Omega-3 fatty acid supplementation, most frequently from fish oil, has emerged as a possible treatment or prevention strategy for MDD in non-pregnant individuals, and may have beneficial effects in pregnant women. Although published observational studies in the psychiatric literature suggest that maternal docosahexaenoic acid (DHA) deficiency may lead to the development of MDD in pregnancy and postpartum, there are more intervention trials suggesting clinical benefit for supplementation with eicosapentaenoic acid (EPA) in MDD.

**Methods/Design:**

The Mothers, Omega-3 and Mental Health study is a double blind, placebo-controlled, randomized controlled trial to assess whether omega-3 fatty acid supplementation may prevent antenatal and postpartum depressive symptoms among pregnant women at risk for depression. We plan to recruit 126 pregnant women at less than 20 weeks gestation from prenatal clinics at two health systems in Ann Arbor, Michigan and the surrounding communities. We will follow them prospectively over the course of their pregnancies and up to 6 weeks postpartum. Enrolled participants will be randomized to one of three groups: a) EPA-rich fish oil supplement (1060 mg EPA plus 274 mg DHA) b) DHA-rich fish oil supplement (900 mg DHA plus 180 mg EPA; or c) a placebo. The primary outcome for this study is the Beck Depression Inventory (BDI) score at 6 weeks postpartum. We will need to randomize 126 women to have 80% power to detect a 50% reduction in participants' mean BDI scores with EPA or DHA supplementation compared with placebo. We will also gather information on secondary outcome measures which will include: omega-3 fatty acid concentrations in maternal plasma and cord blood, pro-inflammatory cytokine levels (IL-1β, IL-6, and TNF-α) in maternal and cord blood, need for and dosage of antidepressant medications, and obstetrical outcomes. Analyses will be by intent to treat.

**Discussion:**

This study compares the relative effectiveness of DHA and EPA at preventing depressive symptoms among pregnant women at risk.

**Trial registration:**

Clinical trial registration number: NCT00711971

## Background

Major depressive disorder (MDD) is prevalent among women of childbearing age. Major depressive disorder is a significant cause of morbidity and mortality in the developed world, affecting 20-25% of women at least once during their lifetime[1]. The primary risk factors for MDD include female gender, family history of depression, and previous personal history of depression[2]. The World Health Organization estimates that MDD is among the top seven causes of disability-adjusted life years worldwide [3-5]. In a 30-year projection of mortality and disability by cause, Murray and Lopez found MDD to be a leading cause of disability-adjusted life years (years of potential life loss due to premature mortality plus years of productive life lost due to disability) for women globally[4].

Depressive symptoms sufficient to disrupt functioning have been estimated to occur in 5-25% of women after delivery [6]. A cross-national review of the literature found the prevalence rates of postpartum depression to vary between 0.5% and 24.5%[7]. A cohort study of depressed mood during pregnancy and after childbirth, based on testing with the Edinburgh Postnatal Depression Scale (EPDS), found that 9.1% of women scored above the threshold for probable depression at 8 weeks postpartum [8]. Postpartum depression is associated with significant functional impairment and with problems in maternal-infant attachment [9]. Despite the emphasis given to postpartum depression, antenatal depression may occur with an equal or greater prevalence. A recently published systematic review estimating the point prevalence of depression during pregnancy found prevalence rates for depression in the first, second, and third trimesters of pregnancy of 7.4%, 12.8% and 12.0%, respectively [9]. Likewise, a cohort study of 9,028 British women found that the prevalence of depression at 32 weeks gestation was 13.5% and actually exceeded the 9.1% depression prevalence at 8 weeks postpartum[8].

Treatment of depression during pregnancy raises mothers' and physicians' concerns regarding fetal safety. Many mothers ultimately express reluctance to take medications during gestation. Indeed, in a population screening study conducted among pregnant women attending antenatal clinics at our own institution, only 14% of those with a positive depression screen received any formal treatment for depression[10]. Women with a history of postpartum depression in a previous pregnancy have a 25-50% risk of depression recurrence [11]. Complications such as preterm birth, preeclampsia, need for cesarean delivery, and poor pregnancy outcome may be increased in women experiencing depression during pregnancy[12]. Women who experience depression during pregnancy are also at increased risk for persistence or worsening of depression in the postpartum period[9].

Depression during the critical period of mother-infant attachment may also adversely affect child neurodevelopment. In a review of the literature concerning infants of mothers suffering from postpartum depression, Grace and colleagues found that children of mothers experiencing postpartum depression performed less well on cognitive tasks than children on non-depressed mothers at 18 months[13]. Differences in language skills and Piaget's object concepts tasks were noted in particular and these effects were more pronounced in boys than in girls. In addition, the authors found that postpartum depression exerted a modest effect on child behaviors up to 5 years of life, including distractibility, and antisocial and neurotic behavior at home and in school[13].

Selective serotonin uptake inhibitors (SSRIs) are the standard treatment for antenatal and postpartum depression and are widely prescribed during and after pregnancy[14]. Some, but not all, observational studies have noted an increased risk for congenital cardiac malformations or pulmonary hypertension with this use of these medications[15]. SSRIs readily cross the placenta; Hendrick *et al *demonstrated that antidepressant and metabolite concentrations were detectable in 87% of cord blood samples of infants of mothers who had received SSRIs [16]. Although neonatal blood levels were below maternal drug levels, the effects of these compounds on the developing brain remain unknown. In a prospective study of newborn neurobehavior, Zeskind demonstrated that newborns that had been exposed to SSRIs in utero had increased motor activity and tremulousness when compared to unexposed neonates [17]. Exposed neonates also exhibited fewer changes in behavioral states and fewer different behavioral states. Changes in the proportion of rapid eye movement sleep were also noted. In another study of infants born after SSRI exposure in utero, Oberlander and colleagues observed that neonatal acute pain response was altered by exposure to SSRIs [18]. Although these observations are of uncertain clinical significance, they indicate that neurobehavioral changes are associated with SSRI exposure in utero [19].

In a meta-analysis of available studies on SSRI use during pregnancy and effects on the fetus and newborn, Lattimore et al found that infants exposed to SSRIs in utero are more likely to be low birth weight and more likely to be admitted to a SCN/NICU at birth than infants born to mothers not on these medications[20]. Sanz and colleagues have analyzed a database of reports of neonatal withdrawal symptoms [21]. Paroxetine was associated with neonatal convulsions and cases of neonatal withdrawal were reported after exposure to all SSRI drugs. Based upon these data, Ruchkin and Martin recommended that the threshold for prescribing these compounds during pregnancy and in the postpartum should be raised[22]. Concerns about these compounds have also led to an interest in complementary and alternative methods of preventing or treating MDD.

Omega-3 fatty acids, most frequently from fish oil, are one such complementary and alternative medicine method. There is a heterogenous literature surrounding the use of omega-3 fatty acids for mood disorders. Published systematic reviews of omega-3 fatty acids for monotherapy or adjunctive therapy of MDD in non-pregnant populations have reached conflicting conclusions regarding efficacy and called for further research[23-25]. While systematic reviews of trials of omega-3 fatty acids in affective disorders by Freeman, [23] Lin, [25] and Appleton, [26] found that supplementation produced statistical improvement in mood symptoms, these authors' analyses found considerable between-study heterogeneity, possibly due to differences in the populations studied, timing of administration, and differing proportions of the two main omega-3 fatty acids, eicosapentaenoic acid (EPA) and docosahexaenoic acid (DHA) in the fish oils studied.

Likewise, although omega-3 fatty acids might offer an alternative to SSRIs for antenatal and postpartum depression, there has been conflicting evidence of efficacy from the available clinical trials. In a recent systematic review of placebo-controlled trials of omega-3 fatty acids for prevention or treatment of perinatal depression, Jans *et al *did not find benefit for omega-3 supplementation in their total analysis. However, there was some evidence of effectiveness among women with MDD[27]. A recent large placebo-controlled randomized controlled trial (RCT) of DHA for prevention of perinatal depression among pregnant women not selected by risk status did not show a benefit of DHA for this indication[28].

One possible reason for the heterogeneity of results of the available trials may be differing effectiveness of DHA and EPA. Two systematic reviews of published RCT's have found EPA but not DHA to be effective in alleviating depressive symptoms[29,30]. Thus the Omega-3 fatty acids subcommittee assembled by the Committee on Research on Psychiatric Treatments of the American Psychiatric Association has called for further randomized controlled trials of omega-3 fatty acids for mood disorders, and in particular comparisons of EPA and DHA [23]. To our knowledge, the proposed study is the first to directly compare EPA and DHA-rich fish oils in a pregnant population.

## Methods

### Aims

The aim of this study is to determine whether EPA or DHA will prevent pregnancy-related depressive symptoms. We hypothesize that EPA-rich fish oil supplementation and DHA-rich fish oil supplementation will reduce the risk of antenatal and postpartum depressive symptoms in women who are at elevated risk for depression based on a previous history of major depressive disorder, a previous history of postpartum depression, or an Edinburgh Postnatal Depression Scale (EPDS) score between 9 and 19 in early pregnancy. The study will also directly compare the effects of EPA and DHA on depressive symptoms as measured by the Beck Depression Inventory score. In addition we hypothesize that fewer pregnant women assigned to the fish oil supplementation will require treatment with antidepressant medications and if medicated, a lower total dose will be required than for women receiving placebo. We will recruit pregnant women who are between 12-20 weeks gestation and will follow them through their pregnancies and up to 6 weeks postpartum. The women participating in this study will be receiving prenatal care at two health systems in southeastern Michigan, the University of Michigan Hospitals and Clinics and St. Joseph Mercy Health System/Integrated Health Associates. These health systems are located in Ann Arbor, Michigan and Ypsilanti, Michigan and the surrounding communities.

This study also has three secondary aims that will generate preliminary data on possible mechanisms of action of EPA and DHA in mental health. First, we will examine the relationship between EPA and DHA, maternal plasma fatty acid levels, and symptoms of depression. Second, we will explore whether EPA and DHA reduce depressive symptoms by reducing synthesis of pro-inflammatory cytokines IL-6, IL-1β, and TNF-α. Third, we will examine the relationship between EPA and DHA and standard pregnancy outcomes, including birth weight and length of gestation.

### Participants/Eligibility Criteria

To be eligible to participate in the study, women must be 18 years of age or older, have a confirmed, live, intrauterine pregnancy of greater than 12 weeks gestation but less than 20 weeks gestation, and be enrolled in prenatal care at one of the two participating health systems. They must be planning to deliver at one of the two participating hospitals and plan to remain in the southeastern Michigan area through 6 weeks after delivery. To qualify for the study, women must be at risk for depression, based on (i) a history of MDD, (ii) a history of postpartum depression, or (iii) an EPDS score between 9 and 19.

Women will be excluded from participating in the study if they are currently taking an omega-3 fatty acid supplement; taking antidepressant or other psychiatric medications; using therapeutic or prophylactic anticoagulation medication; or consuming more than 2 fish meals per week. In addition, pregnant women with a diagnosis of current major depressive disorder, or other psychiatric diagnoses, including current substance abuse, schizophrenia, and bipolar disorder will be excluded from study. Pregnant women with a history of bleeding disorder such as von Willebrand's Disease will also be excluded.

### Recruitment, Randomization, and Collection of Baseline Data

Screening for depression using the EPDS screen is carried out as standard clinical practice at most, but not all, of the participating clinical care sites. Clinicians caring for pregnant women use the EPDS to identify women at risk and to facilitate mental health referrals. We will identify our eligible population of women with EPDS 9-19 or a past history of MDD through a variety of mechanisms, including health care provider referrals, advertisements, a pregnancy and mental health research registry, and a screening questionnaire and "permission to determine eligibility" form administered at the time of visits to the obstetrician or midwife, or to an obstetrical ultrasound unit.

The EPDS is a widely used, valid and reliable 10 item measure of perinatal mood[31]. This scale was chosen because it is less reliant on somatic complaints than other depression scales, and somatic complaints are very common in normal pregnancy. The EPDS is self-administered and may be scored by a wide range of health care workers. The EPDS has also been shown to be responsive to mood changes as a function of treatment[32]. A cut off score of 12 has been recommended for detecting the presence of MDD, with sensitivities found to be .68 - 1.0 and specificities of .49-1.0[33]. EPDS scores above 9 may indicate subclinical depression and risk for later development of MDD[34].

Those eligible women who agree to be contacted will be given information about the study, and offered study participation. All eligible women are informed of the risks and benefits of study participation, and reminded that study participation is voluntary. Full written informed consent will be solicited. Women who agree to participate will undergo a final determination of eligibility with administration of the Mini International Neuropsychiatric Interview (MINI), either by phone or in person. The MINI will identify women with current MDD or other major psychiatric diagnoses or substance dependence. These women are not eligible for the study and will be excluded from the sample population prior to randomization. They will be offered referral information for appropriate mental health care. Women meeting all eligibility criteria will be randomized and will attend 4 study visits.

Randomization will be carried out by use of a random number table maintained in the University of Michigan Investigational Drug Service (IDS). The research team will be blinded to the results of the randomization. The research team will provide the IDS staff with a list of unique identifying numbers prior to the start of recruitment and, at the time of randomization, will inform the staff of the study identification number of each participant to be randomized. The IDS staff will then randomize participants to one of three study arms using a random number table: intervention #1, intervention #2, or placebo and will maintain randomization records until the last participant has completed the protocol.

The IDS will dispense all study supplement which will consist of either: a) EPA-rich fish oil supplement (1060 mg EPA plus 274 mg DHA) (intervention #1); b) DHA-rich fish oil supplement (900 mg DHA plus 180 mg EPA) (intervention #2); and c) a placebo (control arm). Because the EPA and DHA capsules are not identical in appearance, all subjects will receive some placebo capsules. For the EPA group, this will involve taking 2 large EPA capsules and 4 small placebo capsules daily; the DHA group will take 2 large placebo capsules and 4 small DHA capsules daily. The placebo group will take 2 large placebo capsules and 4 small placebo capsules daily.

The intervention supplements and placebo have been provided by *Nordic Naturals *in Watsonville, California. The EPA-rich fish oil (*ProEPAXtra*, *Nordic Naturals*, Watsonville, CA) planned as intervention #1 in this study contains an approximate 4:1 ratio of EPA to DHA (1060 mg EPA plus 274 mg DHA). The DHA-rich oil (*ProDHA*, *Nordic Naturals*, Watsonville, CA) to be studied as intervention #2 contains DHA and EPA in an approximate 4:1 ratio (900 mg DHA plus 180 mg EPA). We chose these fish oil supplements because of their EPA-rich and DHA-rich compositions. The control group (intervention #3) will receive a placebo that will contain 98% soybean oil and 1% each of lemon and fish oil and will be indistinguishable in appearance and taste from the active supplements. The supplements will be molecularly distilled and free of industrial contaminants, mercury, and organochlorines.

### Study Visits

As much as possible, the four study visits will be coordinated with the subjects' routine prenatal care or ultrasound appointments. Visit #1 will occur between 12 and 20 weeks' gestation. Full written informed consent will be obtained. Baseline demographic and past medical and obstetrical history data will be collected. The baseline BDI (Beck Depression Inventory) will be administered. After a 3-hour fast, subjects will have their blood drawn to measure their baseline omega-3 fatty acid and proinflammatory cytokine levels (IL-1β, IL-6, and TNF-α). Subjects will be given a 3 month supply of the study supplement at this visit. Subjects will be instructed to take three capsules twice daily with meals and to return all unused capsules to the research team at their next study visit.

The second study visit will take place between 26 and 28 weeks gestation. During this visit, the BDI will be completed and the MINI will be re-administered to assess for MDD, suicidal ideation and psychiatric co-morbidity. The subjects will return their prescriptions to the study staff and all unused capsules will be counted. A two month supply of the study supplement will be given to each subject.

The third study visit will take place between 34 and 36 weeks gestation. During this morning visit, the BDI will be completed and the MINI will be re-administered. In addition, a second fasting blood sample will be obtained to measure the participant's omega-3 fatty acid levels and proinflammatory cytokine levels (IL-1β, IL-6, and TNF-α). At this visit, a three-month supply will be dispensed in order to provide women with sufficient doses until the postpartum visit. Each participant will be provided with a packet containing sterile tubes for collection of cord blood. Participants will be instructed to bring this packet with them to the hospital when they present to the Labor and Delivery unit.

When the subject presents for delivery, clinical staff will be notified that the patient is a study participant. After delivery of the baby, cord blood will be collected and proinflammatory cytokine levels and fatty acid levels will be assayed (visit #4).

The final study visit will take place at the participant's 6 week postpartum check-up. During this visit, the BDI will be completed and the MINI will be re-administered. Participants will receive $20 and a parking voucher for each study visit that they attend.

Capsule compliance will be monitored by pill counts. Adherence to the study protocol will also be ascertained by measurement of omega-3 fatty acid concentrations in maternal plasma at baseline and at 34-36 weeks' gestation.

Study participants who score above at or above a 20 on the BDI or who meet criteria for MDD on the MINI at any time during the study period will be referred for appropriate management with a mental health provider, who may prescribe antidepressant medications if indicated. In this case, the participant will be instructed to continue with the study intervention during treatment and to complete all study visits. Any subject expressing suicidal ideation will be escorted to the psychiatric emergency department.

## Results/Outcomes

### Primary Outcome Measure

The primary outcome of interest in the proposed study is the participant's BDI score at 6 weeks postpartum. The BDI is widely accepted and will be used to measure the severity of depressive symptoms during pregnancy and in the postpartum period. It was first introduced by Dr. Aaron Beck and colleagues in 1961 (BDI-I) and consists of 21 questions about how the participant was feeling in the last week[35]. Each set of at least four possible answer choices range in increasing intensity. The questions touch on depressive symptoms such as hopelessness and irritability, cognitions such as guilt or feelings of being punished, as well as physical symptoms such as fatigue, weight loss, and lack of interest in sexual intercourse.

The test characteristics of the BDI as a depression screen among pregnant populations have been well-characterized[36,37]. In a longitudinal study among 105 pregnant women attending an urban antenatal clinic, Holcomb found the BDI to be a rapid and sensitive screening test for depression[37]. The standard cutoffs for the BDI are as follows: 0-9 for no significant depressive symptoms; 10-19 for mild to moderate depression; 20-29 for moderate to severe depression; and 30-63 for severe depression. Higher total scores indicate more severe depressive symptoms. The BDI has been used widely in clinical research and especially in pregnant populations[37-39] and has high internal consistency[40]. The questionnaire takes no more than 10 minutes to complete and is prepared at a 5^th ^to 6^th ^grade reading level.

For the proposed study, the BDI will be administered at baseline, 26-28 and 34-36 weeks gestation, and at 6 weeks postpartum. As our primary outcome of interest, depressive symptoms will be measured as a continuous variable and reported in descriptive and multivariable analyses as means and standard deviations.

### Secondary Outcomes

Secondary outcomes we will evaluate include a) the need for depression-related treatment and antidepressant medication during and after pregnancy; b) depressive symptoms, as measured by the BDI, at 26-28 and 34-36 weeks gestation; c) changes in omega-3 fatty acid concentrations in maternal plasma at baseline (<20 weeks gestation) and at 34-36 weeks gestation; d) changes in levels of proinflammatory cytokines (IL-1β, IL-6, and TNF-α ) at baseline and at 34-36 weeks gestation; and e) standard obstetric outcomes including length of gestation, birth weight, Apgar score, mode of delivery, blood loss at delivery, gestational hypertension, gestational diabetes, and preeclampsia. Neonatal outcomes, including cord umbilical pH, length of stay, NICU admission, and antibiotic administration will be recorded as well. We will also evaluate the effect of maternal omega-3 fatty acid supplementation on cord blood fatty acid levels as well as fetal synthesis of proinflammatory cytokines IL-6, IL-1β, and TNF-α.

Information on pregnancy outcomes will be extracted from the electronic medical record. Preterm birth will be defined as delivery before 37 completed weeks' gestation and post-term gestation will be defined as delivery at or beyond 42 completed weeks' gestation. Birth weight, Apgar scores, mode of labor onset (spontaneous or induced), mode of delivery, and estimated blood loss at delivery are all available in the electronic record. Blood loss at delivery is routinely estimated by the delivering physician or midwife. Gestational hypertension will be defined as systolic blood pressure (BP) elevation ≥30 mm Hg or diastolic BP elevation >15 mm Hg compared to early pregnancy BP. Preeclampsia will be defined by blood pressure criteria noted above plus >1+ proteinuria on urine dipstick or > 300 mg protein on 24 hour urine collection. Gestational diabetes will be diagnosed based on a 1 hour glucose after a 50 gm glucose load of ≥200 or 2 abnormal values on a 3 hour glucose tolerance test.

### Sample Size Considerations

We hypothesize that (i) fish oil supplementation will reduce the severity of depressive symptoms in the postpartum period among women at risk and that the (ii) EPA-rich fish oil (intervention #1) and DHA-rich fish oil (intervention #2) will be equally efficacious in reducing depressive symptoms. Our sample size calculation is based on the premise that fish oil supplementation of either the EPA-rich or DHA-rich dose will result in a 50 percent reduction in the mean BDI score at 6 weeks postpartum. Sample size calculations were generated in *nQuery Advisor *Version 6.01 based on data from a previous study conducted among the target population by one of the co-investigators, Dr. Delia Vazquez. In that observational study, she observed a mean BDI score of 8.4 with a standard deviation of 6.4 among postpartum mothers at risk for depression. Assuming a significance level of 0.05, a one-way analysis of variance test, a variance of means (variance of the individual group means) of 3.920, a standard deviation of 6.4, and an effect size (the index of the separation expected among the observed means) of 0.0957, we will need to recruit 105 pregnant women (35 pregnant women in each of the three groups) to have 80% power to detect at least one group difference in the mean BDI score. We propose to recruit 126 pregnant women to account for the possibility of up to 10% study dropout and 10% initiating antidepressant therapy during the study period (to provide sufficient numbers for subgroup analyses).

Our decision to hypothesize a 50% reduction in the mean BDI score between intervention and control groups is based on results from published studies. Freeman and colleagues recently conducted two clinical trials[41,42] of omega-3 fatty acid supplementation and perinatal depression, using the EPDS as their primary measure of depressive symptomatology and reported a 51% and 41% reduction in EPDS score over an 8 week study period. A comparable study in a non-pregnant population demonstrated a similar effect size[43].

### Analysis Plan

We propose to recruit 126 women to examine the effect of a prenatal omega-3 fatty acid supplementation intervention on the risk of postpartum depressive symptoms at 6 weeks postpartum. Data will be prospectively collected via in-person interview, blood samples for serologic testing, and electronic medical chart abstraction. Information obtained from in-person interview and from chart abstraction will be initially completed by paper by a member of the research team and then transferred to a customized study database. The data files will be password protected and kept in a secure location on our network server. All statistical analyses will be performed using *Statistical Analysis Software (SAS) *Version 9.1 (SAS Institute, Inc., Cary, NC).

### Covariate Assessment

Other experiences of the participant that might influence depressive symptomatology must be considered as potential confounders. These include other exposures that occur during the prenatal and postpartum period. In addition to detailed information on the intervention and outcomes of interest, we will collect a detailed demographic, behavioral, dietary, and medical history from each participant at the time of study enrollment and review the participant's medical record and document any complications incurred during pregnancy.

### Descriptive Analyses

Once data have been collected, descriptive statistics (means, standard deviations, frequencies, proportions, and graphical displays) will be computed for all study variables. The distributions of outcome variables will be assessed to determine if distributional assumptions are valid. Decisions will be made at this time about the creation of category boundaries for certain continuous covariates, using well-established cut-points where appropriate. All analyses will be done on an intent-to-treat basis. Baseline analyses will be performed to determine if the three study groups are comparable or not with respect to various variables including age, parity, and body weight. If any of the baseline variables are shown to be different across the three groups, any outcome comparisons across the three groups will be adjusted for the potential confounding effect of these variables. In all analyses, we plan to control for possible confounders, and covariates that change the coefficient of interest by more than 10% will be retained in the final multivariable model.

### Multivariable Analyses

To assess the effectiveness of a prenatal omega-3 fatty acid supplementation intervention in reducing the incidence and severity of depressive symptoms during pregnancy and up to 6 weeks postpartum, a one-way analysis of variance (ANOVA) with repeated measures will be used via the MIXED procedure in SAS. A one-way ANOVA has the advantage of testing whether there are any differences between the three groups with a single probability associated with the test. The hypothesis tested is that all groups have the same mean. We will have one within factor (time) and one between factor (supplement group). The ANOVA will allow us to assess the significance of the two main effects as well as if interaction is present, adjusting for potential confounding variables. Moreover, as a repeated measures design, it will allow us to use all four BDI assessments, which in turn will provide more detailed information about the trends over time than one or two assessments would. Lastly, we will be able to look at intermediate outcomes of interest, such as the effect of the prenatal omega-3 fatty acid supplementation intervention during pregnancy, using only the three BDI assessments performed during the prenatal period in our one-way ANOVA with repeated measures model.

Women who initiate antidepressant therapy during the study period will remain in the study for its duration. However, it will be necessary to take this change in treatment into account in our multivariable analyses. Thus, in addition to the above mentioned analyses, we will also perform a subgroup analysis among those who were not taking antidepressants during the study period. We will also test whether therapy could be a confounder or effect modifier of the effect of fish oil supplementation on depressive symptomtaology over the entire study cohort.

All multivariable analyses will be performed using the MIXED procedure in SAS, as this procedure allows for missing data. While the research team will make every effort to collect data from all participants at all four time points during the study period, it is possible that a participant might miss one or two study visits. The methodological approach chosen for this proposal allows for some flexibility with the analyses performed. For example, comparisons will be drawn between baseline and 6 week postpartum BDI scores solely, as well as after taking into account the assessments drawn at 26-28 and 34-36 weeks gestation. Unlike the general linear models (GLM) procedure in SAS, the MIXED procedure does not remove all data for any subject that does not have complete data. Rather, all study participants are retained in the analysis and all available information is used.

### Other Planned Exploratory Analyses

To evaluate the effect of fish oil supplementation on omega-3 fatty acid concentrations in maternal blood, we will use a mixed effect model for longitudinal data because the omega-3 fatty acid concentrations will be measured twice for each participant (at baseline and at 34-36 weeks gestation). Using this model, we can assess both the time averaged difference in the concentration between the three groups as well as the change in the concentration over the two measurement times between the three groups. To evaluate the effect of fish oil supplementation on the proinflammatory cytokines referenced, we will use a mixed effect model for longitudinal data because each proinflammatory cytokine level will be measured twice for each participant (at baseline and at 34-36 weeks gestation). Using this model, we can assess both the time averaged difference in the levels between the three groups as well as the change in concentration over the two measurement times between the three groups. To evaluate the effect of fish oil supplementation on standard obstetrical outcomes including but not limited to length of gestation, birth weight, Apgar score, mode of delivery, blood loss at delivery, and preeclampsia, we will use multiple regression for continuous outcomes variables and logistic regression for dichotomous outcome variables, and in either of these approaches, an indicator for fish oil supplementation will be the primary independent variable.

The exploratory aims are intended to generate preliminary data elucidating the possible mechanism of action of fish oil supplementation in the prevention of depression in pregnancy and the postpartum period. In particular, the role of fish oil in inhibiting production of proinflammatory cytokines may provide an explanation for the observed link between depression in pregnancy and preterm birth, a condition known to be associated with inflammation. Although we will be unable to definitively explore these relationships in the proposed study, we intend for these aspects of the study design to generate preliminary data which will allow us to refine our dosing, expected effect sizes, and hypotheses for a larger, multicenter study of the effectiveness of fish oil in prevention of major depression in pregnancy and postpartum.

## Discussion

The proposed study will assess the effect of two fish oil supplements on depressive symptomatology in an at-risk population through a randomized controlled design. Strengths to this study include its double-blind, placebo-controlled design and its inclusion of two intervention groups to test both an EPA-rich and a DHA-rich supplement. To our knowledge, the proposed study is the first to directly compare these two fish oils in a pregnant population. Results of previous systematic reviews indicate that differences in fish oil composition may account for the heterogeneity of the observed results of prevention and treatment trials for depression[29,30]. However, this hypothesis has not been previously tested in a RCT comparing both interventions.

The proposed study has the potential to impact clinical care for pregnant women at risk for depression during and after pregnancy. There is a significant burden of disease associated with major depression, and although depression during and following pregnancy occurs at a frequency similar to preeclampsia (5-8%) and preterm birth (11%), it receives much less clinical attention. The disability associated with depression is as great as the disability of other chronic diseases such as hypertension, diabetes, and rheumatoid arthritis[44]. Maternal depression during pregnancy may also constitute a risk factor for suboptimal neurobehavioral development of the child. Dietary supplementation with molecularly distilled omega-3 fatty acid preparations may provide a safe and well-tolerated means for pregnant women to reduce their risk for depression.

## Abbreviations

MDD: major depressive disorder; DHA: docosahexaenoic acid; EPA: eicosapentaenoic acid; EPDS: Edinburgh Postnatal Depression Scale; BDI: Beck Depression Inventory; SSRI: selective serotonin reuptake inhibitors; IL: interleukin; TNF: tumor necrosis factor; IDS: Investigational Drug Service; MINI: Mini International Neuropsychiatric Interview; UM: University of Michigan; ANOVA: One-way analysis of variance; RCT: randomized controlled trial; BP: blood pressure.

## Competing interests

The authors declare that they have no competing interests.

## Authors' contributions

This study was designed by EM, AV, ZD, SM, and DV. EM is the principal investigator for this study. KK is the principal investigator for the St. Joseph Mercy Hospital site. AV is the project statistician. JC is the study coordinator. SM has provided psychiatric oversight for all aspects of screening and study participation. CK, SH, LA, and JC have participated in subject recruitment, administration of the Mini International Neuropsychiatric Interview, and meeting with subjects at each of the four study visits described in this protocol. EM and DB met with subjects at study visits. EM and AV, as well as JC and LA wrote and edited this manuscript. All authors read and approved the final version of this manuscript.

## Details of Ethics Approval

This study has been approved by the institutional review boards of the University of Michigan Health System and the St. Joseph Mercy Health System. This trial is registered with the Cochrane Clinical Trials Registry and at: http://clinicaltrials.gov/ct2/results?term=Mozurkewich The study is being carried out under a United States Food and Drug Administration (FDA) Investigational New Drug Application for the supplements under study: *ProEPA Xtra*, and *Pro DHA*. There is a three person data safety monitoring committee (DSMC) that meets quarterly. Current members of the DSMB are Drs. Barbara Luke, Charles Neal, and Dwight Rouse. Dr. Mel Barclay served on the committee until his death in 2010.

## Pre-publication history

The pre-publication history for this paper can be accessed here:

http://www.biomedcentral.com/1471-2393/11/46/prepub
